# Increased levels of cortisol are associated with the severity of experimental visceral leishmaniasis in a *Leishmania (L.) infantum*-hamster model

**DOI:** 10.1371/journal.pntd.0009987

**Published:** 2021-11-23

**Authors:** Tayany de D. Barros-Gonçalves, Andrea F. Saavedra, Luzinei da Silva-Couto, Raquel P. Ribeiro-Romão, Milla Bezerra-Paiva, Adriano Gomes-Silva, Vinicius F. Carvalho, Alda Maria Da-Cruz, Eduardo F. Pinto

**Affiliations:** 1 Laboratório Interdisciplinar de Pesquisas Médicas, Instituto Oswaldo Cruz, FIOCRUZ, Rio de Janeiro, Brazil; 2 Instituto Nacional de Infectologia Evandro Chagas, FIOCRUZ, Rio de Janeiro, Brazil; 3 Laboratório de Inflamação, Instituto Oswaldo Cruz, FIOCRUZ, Rio de Janeiro, Brazil; 4 Instituto Nacional de Ciência e Tecnologia em Neuroimunomodulação (INCT-NIM), CNPq, Rio de Janeiro, Brazil; 5 Disciplina de Parasitologia-DMIP, Faculdade de Ciências Médicas, UERJ, Rio de Janeiro, Brazil; 6 Rede de Pesquisas em Saúde do Estado do Rio de Janeiro/FAPERJ, Rio de Janeiro, Brazil; US Food and Drug Administration, UNITED STATES

## Abstract

**Background:**

Several infectious diseases are associated with hypothalamic-pituitary-adrenal (HPA) axis disorders by elevating circulating glucocorticoids (GCs), which are known to have an immunosuppressive potential. We conducted this study in golden hamsters, a suitable model for human visceral leishmaniasis (VL), to investigate the relationship of *Leishmania (L*.*) infantum* infection on cortisol production and VL severity.

**Methods:**

*L*. *infantum-*infected (n = 42) and uninfected hamsters (n = 30) were followed-up at 30, 120, and 180 days post-infection (dpi). Plasma cortisol was analyzed by radioimmunoassay and cytokines, inducible nitric oxide synthase (iNOS), and arginase by RT-qPCR.

**Results:**

All hamsters showed splenomegaly at 180 dpi. Increased parasite burden was associated with higher arginase expression and lower iNOS induction. Cortisol levels were elevated in infected animals in all-time points evaluated. Except for monocytes, all other leucocytes showed a strong negative correlation with cortisol, while transaminases were positively correlated. Immunological markers as interleukin (IL)-6, IL-1β, IL-10, and transforming growth-factor-β (TGF-β) were positively correlated to cortisol production, while interferon-γ (IFN-γ) presented a negative correlation. A network analysis showed cortisol as an important knot linking clinical status and immunological parameters.

**Conclusions:**

These results suggest that *L*. *infantum* increases the systemic levels of cortisol, which showed to be associated with hematological, biochemical, and immunological parameters associated to VL severity.

## Author summary

Visceral leishmaniasis (VL) is an infectious disease that is common in most tropical countries. VL has high morbidity and leads to death if not properly treated. In Brazil, *Leishmania (Leishmania) infantum* is the main causative agent of VL. Golden hamsters have proven to be a suitable model for VL. Despite the importance of hypothalamic-pituitary-adrenal (HPA) axis disturbances in infectious disease, few studies have addressed this issue in VL. In this study, we showed that *L*. *infantum*-infected hamsters present augmented levels of plasmatic cortisol in association with increased spleen parasite burden. Indeed, a strong positive correlation was observed between cortisol and biochemical parameters (AST/ALT/ALP) related to liver damage, as well as pro-inflammatory cytokines (IL-6 and IL-1β), anti-inflammatory cytokines (IL-10 and TGF-β), and the arginase enzyme that may favor the progression of infection. On the other side, cortisol was negatively correlated with leucocytes, except monocytes, and with IFN-γ and iNOS, which are involved in parasite-killing macrophage function. These results shed light on an unexplored aspect of VL pathogenesis, which is the importance of cortisol production in the disease-associated immune dysfunction.

## Introduction

Visceral leishmaniasis (VL), also known as kala-zar, is a neglected tropical disease caused by protozoa of the *Leishmania donovani* complex [1]. An estimated 50,000 to 90,000 new cases of VL occur worldwide each year [2]. In Latin America, cases of VL are reported in at least 12 countries where 63,331 new cases have been registered from 2001 to 2018, with an average of 3,518 cases per year. Most of these (97%) were registered in Brazil [3], where *L*. *(L*.*) infantum* species is the causal agent [4]. Once infected, the host can maintain asymptomatic or evolve to a broad spectrum of clinical manifestations, more commonly hepatosplenomegaly, fever, weight loss, and anemia. The disease can have a progressive fate, evolving as a systemic inflammatory response syndrome, which is threatful to life, if untreated [5].

Interestingly, VL immunopathogenesis involves a suppression of effector immune responses, besides a strong cellular activation, both as consequence of parasite’s antigens induction [6,7]. Bacterial products originated from gut microbial translocation, as lipopolissacaride (LPS), were also recognized as a potentiator factor for the systemic activation mechanisms [8,9]. Consequently, patients present qualitative and quantitative dysfunctions of the effector immune response, disorganization of lymphoid organs systems, and inability to control parasite replication [10,11,12].

The activation of the host’s innate and adaptive immune responses generates a myriad of cytokines, including interleukin (IL)-6, IL-1β, and tumor necrosis factor (TNF), that modulate not only the immune system but also is involved in hypothalamic-pituitary-adrenal axis (HPA) activation [13] with consequent cortisol release. Disorders of the HPA axis activity are reported in several infectious diseases as malaria, Chagas disease, tuberculosis, and viral infections [14,15,16,17] pointing to its relevance for the disease pathogenesis. Evidence of HPA axis disturbances were observed in VL patients, which presented elevated adrenocorticotropic hormone (ACTH) plasma levels, besides normal or moderate increase of cortisol plasma concentration. However, the relationship between hormonal disturbances and clinical and laboratorial features of VL was not evaluated [18,19]. Herein, we conducted studies in *L*. *infantum*-golden hamster (*Mesocricetus auratus*), a suitable animal model for human VL [20]. Our aim was to investigate alterations of the HPA axis during *L*. *infantum* infection by analyzing the cortisol production and its association with the clinical and immunological parameters involved in VL progression.

## Materials and methods

### Ethical approval

This study was approved by the Ethics Committee on Animal Use (CEUA) of Instituto Oswaldo Cruz-FIOCRUZ with protocol number (L-012/2016). The experimental procedures were carried out in strict accordance with the recommendations in the Guide for the Care and Use of Laboratory Animals of the Brazilian National Council of Animal Experimentation (http://www.cobea.org.br).

### Animals

Seventy-two male 6–8 weeks old outbred golden hamsters (*M*. *auratus*) were obtained from animal facilities of the Instituto de Ciência e Tecnologia em Biomodelos (ICTB/FIOCRUZ). The animals were housed in air-conditioned rooms in a controlled environment at temperature with seasonal lighting conditions (12h of light and 12h of darkness), with unrestricted food and water.

### *Leishmania* (*L*.) *infantum* strain and experimental infection

Experiments were performed with the *L*. *infantum* strain (MHOM/PT/88/IMT151). Promastigotes were cultured at 26°C until the stationary phase (fourth day) in Schneider’s medium supplemented [L-glutamine (1mM/mL), antibiotic (penicillin: 200μg/mL, streptomycin: 200μg/mL), and bovine fetal serum (10%)] (Sigma Chemical Co., St. Louis, USA).

Hamsters (n = 42) were infected with 2 x 10^7^ promastigotes of *L*. *infantum* at the stationary growth phase by intraperitoneal (i.p.) route, in a final volume of 50μL. Uninfected hamsters (n = 30) were used as a control group. The infection was monitored for six months, and the animals were euthanized at 30, 120, and 180 days post-infection (dpi), using a combination of anesthetics. Blood was collected by cardiac puncture, between 6:00 and 8:00 a.m. (nadir of the endogenous circadian rhythm) [21]. The blood obtained from each animal was split into three samples: 1) a tube containing heparin for plasma obtention; samples were stored at -20°C for cortisol dosage; 2) a tube containing EDTA for measurement of hematological parameters; 3) a clot activator tube to obtaining serum for biochemical analysis (BD Vacutainer, USA).

### Clinical and parasitological evaluation

Hamsters were monitored during all time points of infection for appearance, swelling, hair loss, weight loss, skin ulceration, and ascites. Animals were weighed before *L*. *infantum* infection and on euthanasia’s days. At each time point, the spleens and livers were removed aseptically, weighted, and analyzed macroscopically based on surface appearance, color, and size. Fragments of each organ were stored for further analysis. The critical point for this study was reached at six months (180 days) after infection.

The liver and spleen parasite burden were quantified by limiting dilution assay (LDA), as previously described [22]. The number of viable parasites per gram of tissue was determined by the mean of the maximum dilution where viable parasites were visualized after 14 days of incubation at 26°C (BOD, São Paulo, Brazil), divided by the weight of the organ fragment. The results were expressed as number of parasites per tissue gram.

### Hematological and biochemical evaluation

The absolute count of red blood cells, hematocrit, hemoglobin, leukocytes, and platelets were quantified in an auto Hematology Analyzer (Sysmex, Kober, Japan). The number of leucocytes per cubic millimeters and the percentages of mono and polymorphonuclear cells were determined by optical microscopy. Biochemical evaluation of serum was performed by dry chemistry system (Johnson & Johnson, New Jersey, USA). The analysis consisted of following tests: alanine aminotransferase (ALT), aspartate aminotransferase (AST), alkaline phosphatase (ALP), total bilirubin (TBIL), albumin (ALB), urea, and creatinine. All the analyses were performed at the Animal de laboratório - análises clínicas Platform, ICTB/FIOCRUZ (PT11-003).

### Quantification of plasmatic cortisol

Plasma cortisol levels were measured by radioimmunoassay (RIA) kits, according to the manufacturer’s specifications (MP Biomedicals, New York, USA). The samples were analyzed in the particle counter WIZARD Automatic Gamma Counter (Perkin Elmer, USA) at the Universidade Federal do Rio de Janeiro—UFRJ.

### Tissue cytokines and enzymes mRNA expression by RT-qPCR

Fragments of spleen were collected in RNA later (Ambion, Life Technologies, Carlsbad, CA, USA) and frozen at −20° C until analysis. Total RNA was extracted using the RNeasy Mini Kit (Qiagen, Austin, Texas, USA) according to the manufacturer’s specifications. The quantification of gene expression was performed for the following RNA gene targets: IFN-γ, TNF, IL-6, IL-1β, IL-10, TGF-β, iNOS, arginase, and the constitutive genes GAPDH and γ-Actin [23]. Relative quantitation of gene expression was calculated using the comparative Ct method (ΔΔCt), with threshold set at 0.02. Gene expression was represented as fold change (2-ΔΔCt) in relation to spleen samples from uninfected hamsters, used as calibrators. The assay was performed on the ViiATM 7 Real-Time PCR System (Applied Biosystems, Foster City, CA, USA) on the PDPT/FIOCRUZ Real-Time PCR Platform RPT09B using the SYBR Green system. Results were expressed in 2^-ΔΔCt^ (fold change).

### Statistical analysis

Comparison between experimental groups was performed using one-way analysis of variance (ANOVA). A parametric or nonparametric test was selected according to the distribution of the raw data, followed by an appropriated post-test (Newman-Keuls test or Dunn’s). Correlation analyses were evaluated by Spearman’s rank test and were performed with GraphPad Prism software version 6.0 for Windows (GraphPad Software, San Diego, CA, USA) or SigmaPlot v12.0 software (Systat Software, Inc). Statistical tests from the ΔCt values were student t-test or Mann-Whitney rank-sum test, and Analysis of Variance.

The results were expressed as median with interquartile range or means ± standard deviation (SD). Heatmap matrix analyses were performed for gene expression ΔΔCt using online software Heat mapper (Wishart Research Group at the University of Alberta). The hierarchical clustering method used for analysis was the average linkage, and the distance measurement method applied was Euclidean. The interaction network was done with correlations that presented significance level p≤0.05 using Cytoscape 3.7.2 software. Values of p *<* 0.05 were considered statistically significant.

## Results

### Clinical and parasitological follow-up of the *Leishmania (L.) infantum* infected hamsters

**Fig 1:**
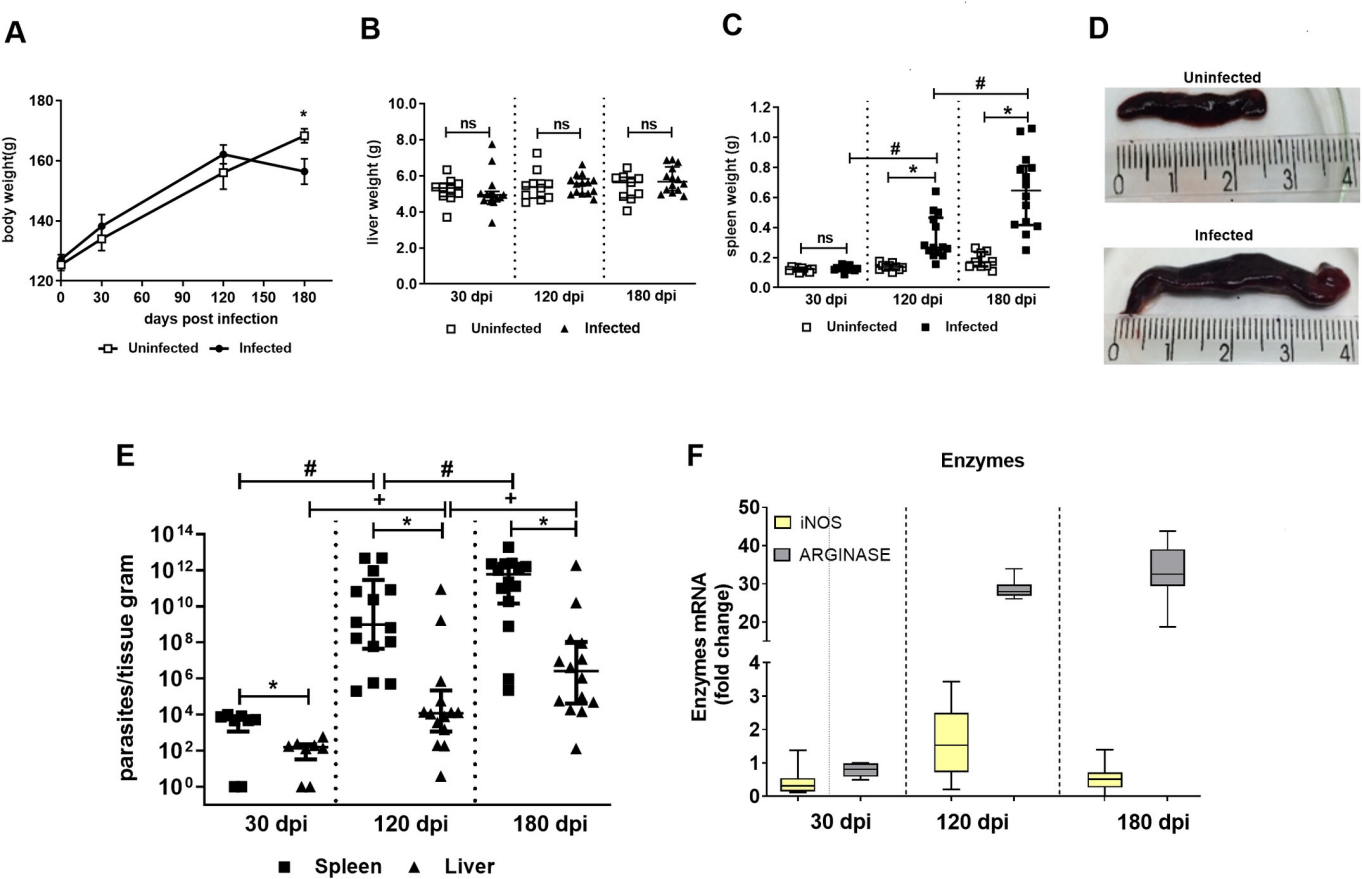
Changes in body, liver and spleen weight, parasite burden of the spleen and liver and gene expression of arginase and iNOS in the spleen of *Leishmania (L.) infantum* infected hamsters. (A) The body, (B) liver and (C) spleen weight of uninfected (n = 10/group) and infected hamsters (n = 14/group) were monitored until 24 weeks post infection (180 days). Infection was made by i.p. route with 2 x 10^7^ promastigotes of *L*. *infantum*. The analysis was made by the ANOVA test. * p< 0.05; ns = not significant. (D) Top photo = uninfected hamster spleen; bottom photo = infected hamster spleen (at 180 day); (E) Parasite burden was quantified by LDA in spleen and liver obtained from infected hamsters (n = 8-14/group), at 30, 120 and 180 dpi. Results of two independent experiments. The analysis was done by the Kruskal Wallis test and Dunns as posttest. * p< 0.05. (F) The relative quantification of mRNA iNOS and arginase was performed by the comparative Ct method (△△Ct), using spleen from uninfected hamster as calibrator (Fold change  =  1), as indicated by the dotted line. Horizontal bars represent the mean ± standard deviation of eight biological replicates.

To evaluate the progression of *L*. *infantum* infection, 14 animals were clinically monitored at 30, 120, and 180 days after the inoculation. Clinical signs suggestive of VL, such as splenomegaly, were observed at 120 dpi (8 animals, 57.1%) and 180 dpi (14 animals, 100%). Ascites was observed in only 3 animals (21.4%). However, weight loss was seen in infected animals at 180 dpi in comparison to the controls (Fig 1A).

No change in liver weight was detected throughout the follow-up of infection (Fig 1B), but liver pallor was observed in 3 animals. Notwithstanding the foregoing, a crescent parasite burden was observed in the liver after 30 dpi over the time of analysis (Fig 1E). Macroscopic changes (splenomegaly) (Fig 1D) and increased spleen weight (Fig 1C) were also observed in infected animals at 120 dpi and most evidently at 180 dpi when compared to uninfected ones. The spleen’ parasite burden was already detected at 30 dpi but increased substantially at 120 dpi and 180 dpi (Fig 1E). The spleen was most parasitized than the liver in all-time points analyzed (Fig 1E). The results showed that even infected with the same inoculum the animals presented a variable clinical behavior.

We also evaluated the spleen gene expression of iNOS and arginase, enzymes involved in both nitric oxide (NO)-mediated parasite killing and polyamine-mediated parasite replication, respectively [24,25]. In accordance with the parasite burden findings, arginase was up-regulated at 120 and 180 dpi (Fig 1F). A small increase in iNOS expression was observed at 120 dpi that returned to basal condition at 180 dpi (Fig 1F).

### Hematological and biochemical changes during *Leishmania (L.) infantum* infection

**Fig 2:**
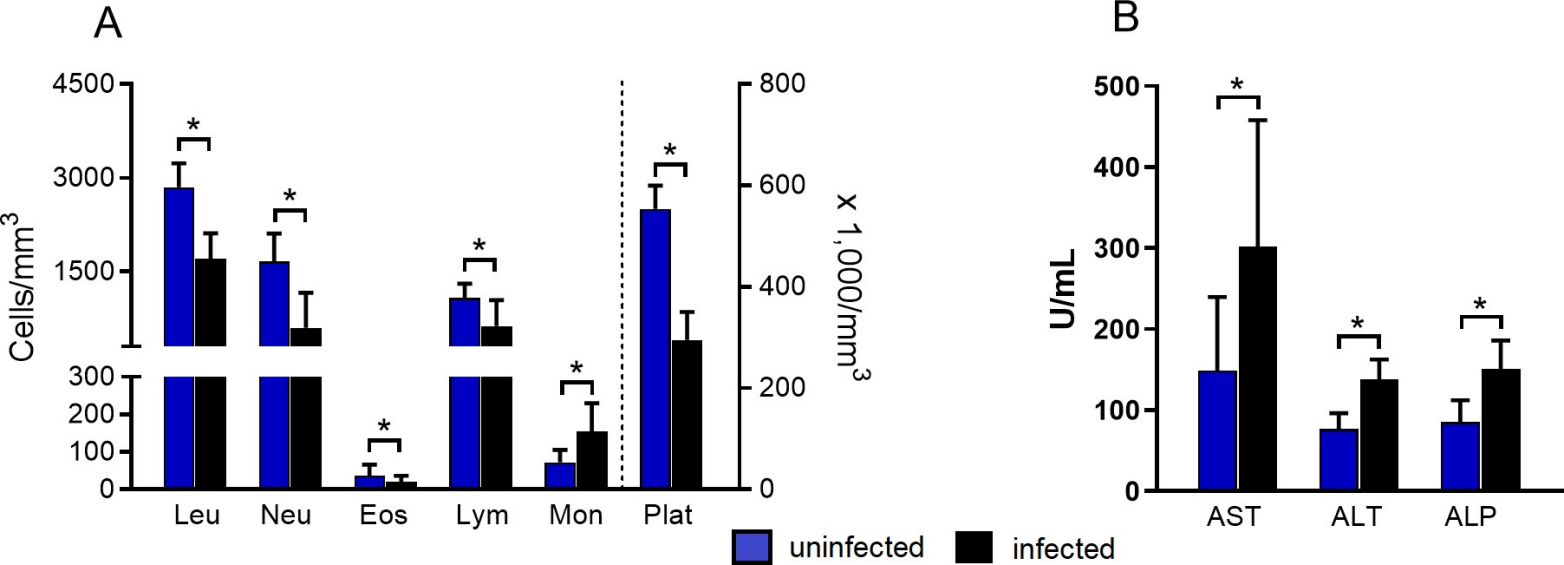
The major hematological and biochemical alterations observed in hamsters at 180 days post *Leishmania (L.) infantum* infection. Left column represented uninfected hamsters (n = 10) and right column represented hamsters infected by *L*. *infantum* (n = 14). Values are represented by mean ± standard deviation; results of two independent experiments. The analysis was done by the Kruskal Wallis test and Dunns as posttest. * p< 0.05. Leu- leukocytes; Neu- neutrophils; Eos- eosinophils; Lym- lymphocytes; Mon- monocytes; PL- platelets; ALT- alanine aminotransferase; AST- aspartate aminotransferase; ASP—alkaline phosphatase.

At 180 dpi, the number of leukocytes was significantly lower in *L*. *infantum-*infected hamsters. Markedly lymphopenia, neutropenia, and eosinopenia were also observed. An increase in monocyte number was seen markedly at 120 dpi. In addition, platelet counts decreased in infected animals in both 120 and 180 dpi (Fig 2A and S1 Table). No alterations on erythrocyte lineage were observed during the evaluation (S1 Table).

The biochemical analysis showed an increase in ALT levels in infected hamsters at 120 and 180 dpi. Higher levels of AST and ALP were also observed in infected animals at 180 dpi, evidencing a liver dysfunction (Fig 2B and S1 Table). No alteration on total bilirubin and albumin levels was seen. No laboratorial alteration compatible with renal dysfunctions was found (S1 Table).

Taking together, we showed a decrease of circulating leucocyte and platelet numbers at 180 dpi compared to uninfected hamsters, but we did not note change in the erythrocyte compartment. Besides, a clear liver and spleen commitment function was presented at 180 dpi.

### *Leishmania (L.) infantum* infected hamsters present increased cortisol plasmatic levels

**Fig 3:**
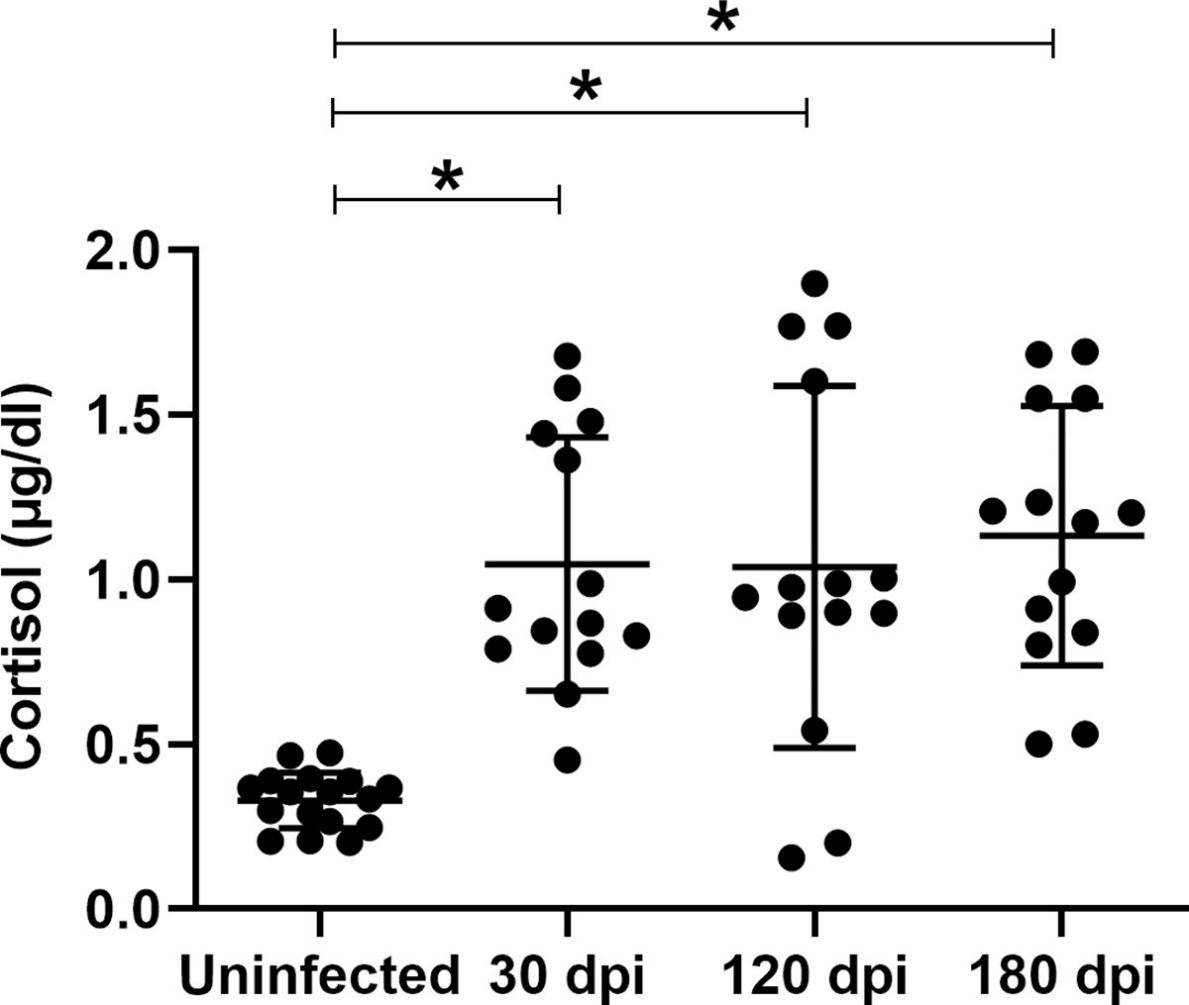
Increase in the systemic levels of cortisol in hamsters infected by *Leishmania (L.) infantum*. Plasma corticosterone were determined at 30, 120 and 180 days post infection by radioimmunoassay in uninfected (n = 17) and infected (n = 14/group) animals. Infection was made by i.p. route with 2 x 10^7^ promastigotes of *L*. *infantum*. Results of two independent experiments. The analyses were done by the ANOVA test and the Newman-Keuls as posttest. *p< 0.05 related to control; ns = not significant. The results were expressed as mean ±standard deviation (SD).

At 30 dpi the *L*. *infantum*-infected animals had high levels of cortisol. These levels maintained elevated at 120 and 180 dpi (Fig 3). Remarkable, there were infected hamsters (varying from 1 to 3 animal/group) presenting cortisol levels lower than 0.5μg/dL, i.e., comparable to the control uninfected group. This demonstrates that adrenal commitment did not occur in all animals, which could be compatible with the variance in the clinical and laboratory behavior observed herein. Then, we would like to investigate whether the cortisol levels had any relationship with the severity parameters of VL.

### Correlation between cortisol production and hematological, biochemical or parasitological parameters

Considering the variability in clinical, parasitological, and laboratory parameters, we decided to evaluate whether they were associated with the range of cortisol levels (Table 1). There was no association with body weight. The spleen and liver parasite burden were positively correlated to cortisol levels at 180 dpi. In accordance with high parasite burden results, spleen arginase expression was positively correlated with cortisol levels, while the correlation with iNOS was negative.

**Table 1:** Correlation between plasma cortisol levels and, clinical, hematological and biochemical variables and gene expression of arginase and iNOS of hamsters infected by *Leishmania (L*.*) infantum*.

Variables	cortisol levels					
	30 dpi		120 dpi		180 dpi	
	*r* ^*a*^	*p* ^*b*^	*r* ^*a*^	*p* ^*b*^	*r* ^*a*^	*p* ^*b*^
Body weight	0.221	0.449	0.477	0.085	0.007	0.982
Liver parasite burden	0.611	0.115	0.738	0.096	**0.569**	**0.034**
Spleen parasite burden	0.467	0.243	0.462	0.085	**0.543**	**0.045**
Arginase mRNA	**0.810**	**0.022**	**0.881**	**0.007**	**0.833**	**0.015**
iNOS mRNA	**-0.881**	**0.007**	**-0.833**	**0.015**	**-0.810**	**0.022**
Hemoglobin	0.316	0.271	-0.304	0.290	-0.317	0,269
Hematocrit	0.126	0.669	0.051	0.864	0.260	0,370
MCV	0.387	0.171	0.196	0.503	0.433	0,122
MCH	0.167	0.568	-0.245	0.398	-0.044	0,880
MCHC	0.425	0.130	-0.458	0.099	0.102	0,729
Leukocytes	-0.471	0.089	-0.323	0.259	**-0.812**	**0.0004**
Basophils	-	-	-	-	-	-
Neutrophils	**-0.670**	**0.009**	**-0.938**	**0.0007**	**-0.780**	**0.001**
Eosinophils	**-0.820**	**0.0003**	**-0.717**	**0.004**	**-0.673**	**0.008**
Lymphocytes	**-0.780**	**0.001**	**-0.899**	**< 0.0001**	**-0.960**	**< 0.0001**
Monocytes	-0.262	0.366	0.002	0.994	0.251	0.387
Platelets	0.090	0.759	-0.327	0.253	0.051	0.864
Urea	0.310	0.281	-0.152	0.605	0.152	0.605
Creatinine	0.124	0.674	0.215	0.460	0.031	0.917
ALT	**0.912**	**< 0.0001**	**0.886**	**0.0003**	**0.793**	**0.001**
AST	**0.969**	**< 0.0001**	**0.938**	**< 0.0001**	**0.899**	**< 0.0001**
ALP	**-**	**-**	**0.807**	**0.0005**	**0.770**	**0.001**
TBIL	-	-	-0.044	0,881	-0.014	0.963
ALB	-	-	-	-	0.464	0.094

Abbreviation: mean corpuscular volume (MCV), mean corpuscular hemoglobin (MCH), mean corpuscular hemoglobin concentration (MCHC), alanine aminotransferase (ALT), aspartate aminotransferase (AST), alkaline phosphatase (ALP), total bilirubin (TBIL), albumin (ALB), days post-infection (dpi).

^a^ Correlation was done using Spearman’s rank and the correlation coefficients are represented by *r* values; n = 14.

^b^ Value of significance *p<0*.*05*.

In general, there was close correlation between cortisol and the laboratory parameters altered during the clinical evolution (Table 1). Interestingly, although the number of total leucocytes decreased only at 180 dpi, we observed a strong negative correlation with lymphocytes, neutrophils, and eosinophils in all-time points evaluated. No correlations were observed for erythrocytes, monocytes, and platelets. Looking at organ function, a strong positive correlation was also found between cortisol and liver enzymes (p<0.001) during all the time points evaluated. The *r* values (varying from 0.770 to 0.969) were remarkably high from all of them: ALT, AST, and ALP. The results indicate that increased release of cortisol during *L*. *infantum* infection can display any role in parasite replication, possibly related to immunological parameters.

### Gene expression of immunological markers in the spleen of hamster infected by *Leishmania (L*.*) infantum*

**Fig 4:**
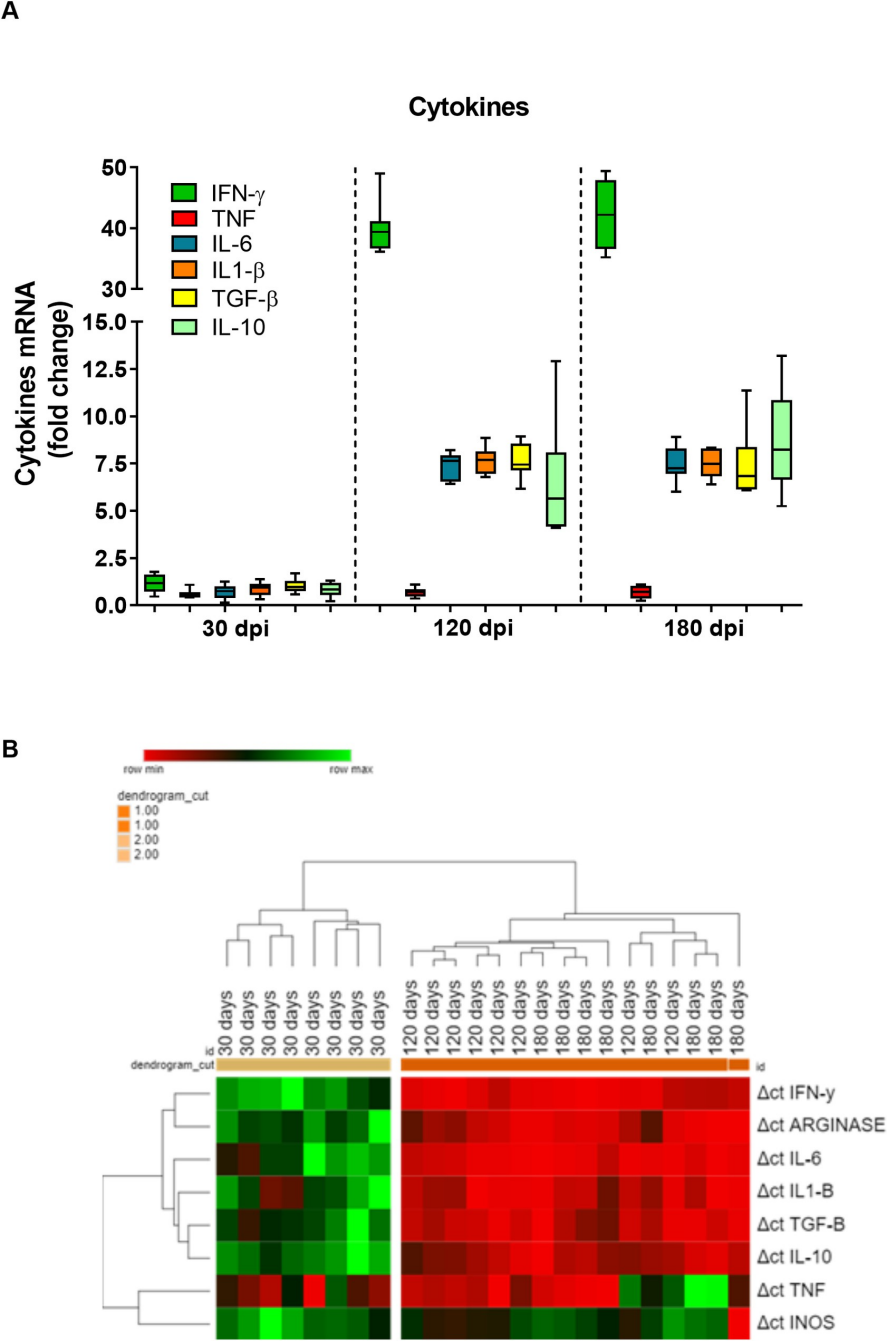
Gene expression of cytokines in the spleen of hamsters infected by *Leishmania (L.) infantum*. A) The relative quantification of mRNA was performed by the comparative Ct method (△△Ct), using spleen from uninfected hamster as calibrator (Fold change  =  1), as indicated by the dotted line. Horizontal bars represent the mean ± standard deviation of eight biological replicates. B) Cytokines, iNOS and arginase in the spleen of hamsters infected with *L*. *infantum* according to a comparative gene expression heatmap analysis. Heatmap analyses were performed for gene expression (*tnf*, *tgfβ*, *ifnγ*, *il10*, *il6*, *il1β*, *arg*, *inos*) ΔΔCt using the online Heat mapper software. The animals were clustered by gene expression signature. The hierarchy average linkage clustering method with Euclidean distance measurement was used (Wishart Research Group at the University of Alberta). Each row represents one molecule, and each column represents one animal. Higher gene expression is displayed in red, and lower gene expression, in green. Gray indicates mean gene expression below the lower limit of detection.

As glucocorticoids can impair immune responses, we analyzed the changes in spleen cytokine commonly related to VL pathogenesis (Fig 4A). Except for TNF, all the anti-inflammatory (TGF-β, IL-10) and pro-inflammatory (IFN-γ, IL-6, IL-1β) cytokines were overexpressed at 120 and 180 dpi, however, no increase of cytokines gene expression was observed at 30 dpi. Interestingly, IFN-γ was highly expressed, around 40-fold over the control groups at 120 and 180 dpi.

We used heatmaps in an attempt to group animals presenting similar spleen cytokine patterns (Fig 4B). Cluster 1 grouped the animals at 30 dpi, whereas cluster 2 animals grouped animals in the late phase of the infection (120 and 180 dpi). The difference between these clusters was the upregulation of arginase and all anti-inflammatory and pro-inflammatory cytokine expressions, except for TNF.

**Fig 5:**
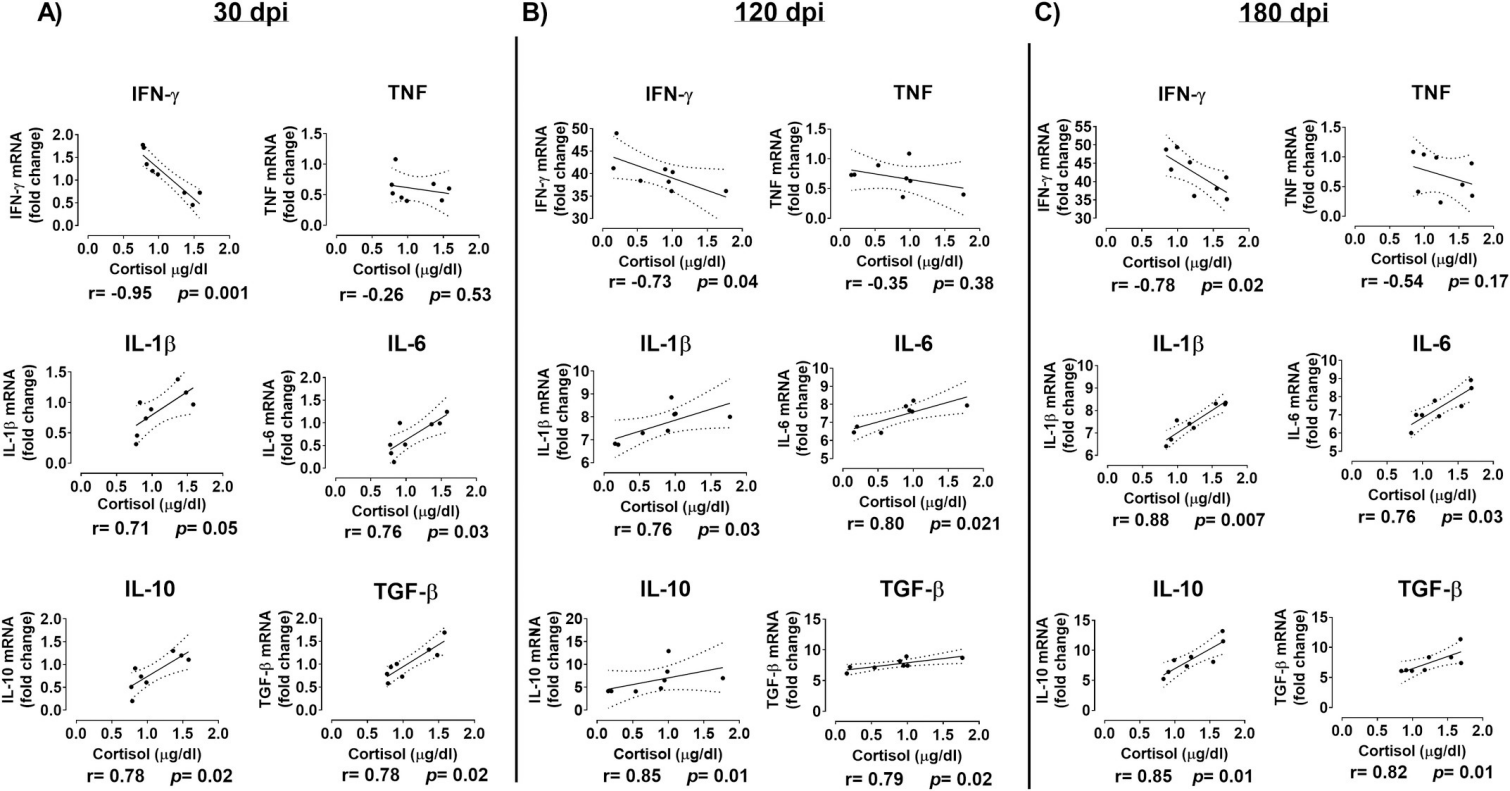
Correlation between cytokines expression and cortisol production in hamsters infected by *Leishmania (L.) infantum*. Cytokine’s expression was detected in the spleen of infected hamsters (n = 8) by RT qPCR and plasma cortisol by RIA. Infection was carried out by i.p. route with 2 x 10^7^ promastigotes of *L*. *infantum*. Spearman’s correlation coefficients are represented by (r values). Values of significance p *<0*.*05*.

IFN-γ expression was negatively correlated with cortisol levels, but no association was seen for TNF. Otherwise, a strong positive correlation with cortisol was observed not only for IL-6, IL-1β but also for IL-10 and TGF-β (Fig 5). The results show that cortisol levels have a close relationship with clinical, parasitological, and immunological evolution of experimental VL.

### Cortisol seems to be an important node in a network involving immunopathological features driving the clinical outcome of experimental visceral leishmaniasis

**Fig 6:**
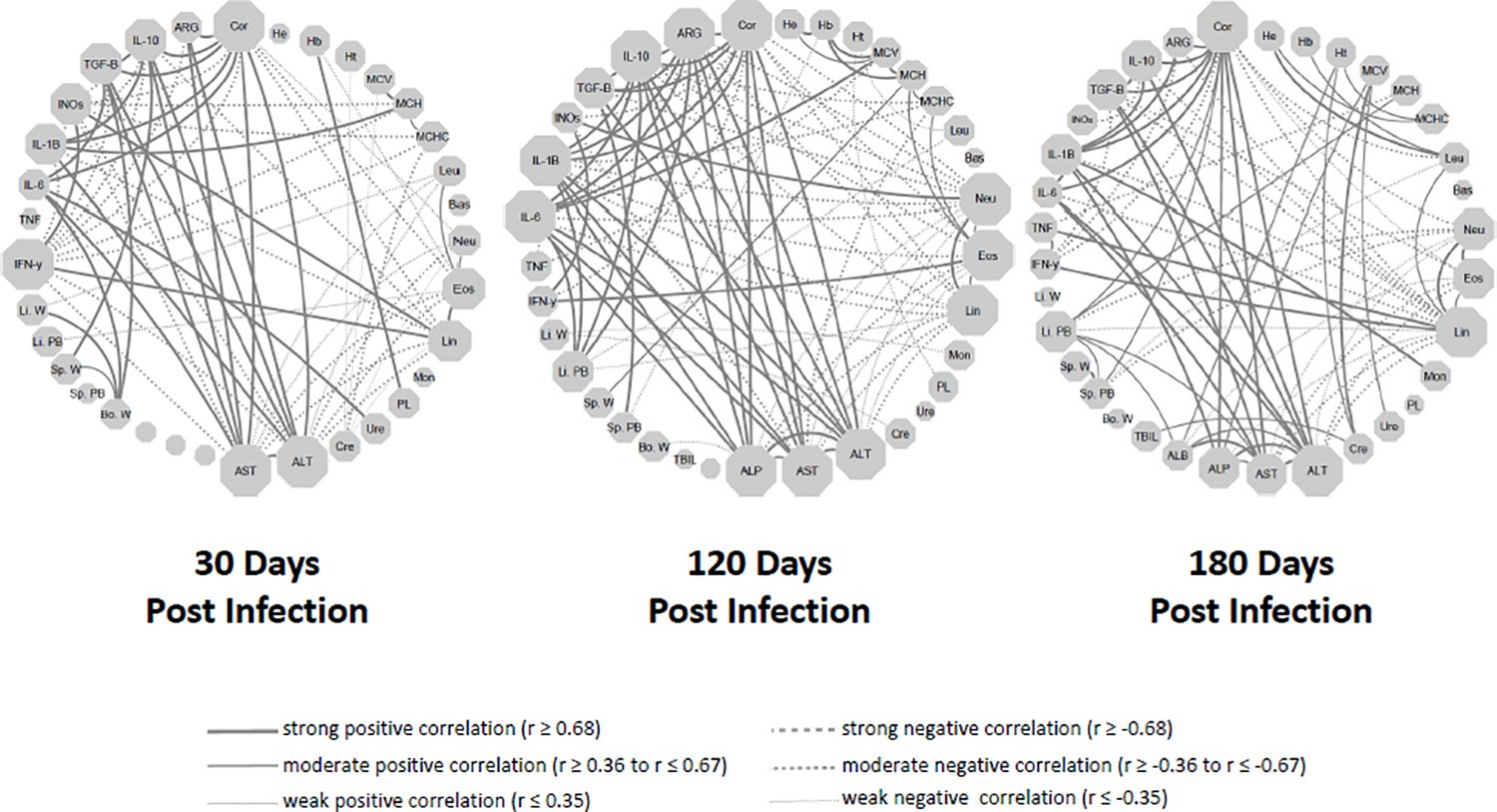
Network interaction involving cortisol production and clinical, parasitological, hematological, biochemical, and immunological biomarkers in hamsters infected with *Leishmania (L.) infantum* along 30, 120 and 180 days. Each node represents a biologic parameter. Significant Spearman correlations (p ≤ 0.05) were represented by continuous lines (positive correlation) or dotted line (negative correlation) connecting the circle nodes. The line thicknesses were directly associated to correlation index and the node size was directly related to the number of interactions. Hb- hemoglobin; Ht- hematocrit; MCV- mean corpuscular volume; MCH- mean corpuscular hemoglobin; MCHC- mean corpuscular hemoglobin concentration; Leu- leukocytes; Bas-basophils; Neu- neutrophils; Eos- eosinophils; Lym- lymphocytes; Mon- monocytes; PL- platelets; Ure- urea; Cre-creatinine; ALT- alanine aminotransferase; AST- aspartate aminotransferase; ASP—alkaline phosphatase; ALB- albumin; TBIL- total bilirubin; Bo. W- body weight; Sp.PB–spleen parasite burden; Sp. W–spleen weight; Li.PB- liver parasite burden; Li.W–liver weight; Cytokine genes–IFN-γ, TNF, IL-6, IL-1β, TGF-β, IL-10; ARG–arginase; iNOS–Oxid nitric sintase.

To globally evaluate the clinical immunopathological status of the disease, a network analysis of Spearman correlations including all studied biological parameters (Fig 6) was carried out.

Along the 30, 120, and 180 days after *L*. *infantum* infection an intense network was established. Positive correlations were observed between the cortisol levels and the gene expression of factors related to promoting visceral leishmaniasis, such as arginase, IL-10, TGF-β, IL-1β, and IL-6. Transaminases associated with liver damage, normally affected by *L infantum* infection, were also positively correlated with cortisol. On the other side, cortisol was negatively correlated with leucocytes, except monocytes, and with IFN-γ and iNOS, which are related to inducing a microbicidal environment. Among the analyzed biomarkers, cortisol together with transaminases defined a signature of high number of interactions that remained throughout the time of infection by *L*. *infantum* in the hamster model.

## Discussion

It is known that the chronic elevation of glucocorticoids can directly influence the impairment of the immune response, being detrimental to the organism [26,27]. However, the neuroimmunoendocrine synergy and disturbance are not well explored in leishmaniasis, especially in VL infection. Here, we investigated the association of HPA axis disorders, measured by the stress-related hormone (glucocorticoids) imbalance with clinical, parasitological, and immunological parameters in *L*. *infantum-*infected hamsters that may lead to VL progression.

The infected hamsters studied here evolved with splenomegaly, ascites, and weight loss compatible with the classic clinical features expected to human and experimental VL [28,29]. The clinical picture varied from asymptomatic to a mild VL diseases as observed by others [30], reproducing differing degrees of severity observed in human VL.

Leishmanial parasites in liver and spleen were observed in all animals, confirming that they were susceptible to *L*. *infantum* infection. The intense spleen parasite replication promotes several modifications, including hyperplasia, loss of specific cell populations, and rupture of splenic architecture, which in turn favored the infection progression [10,30,31].

As observed in *L*. *donovani*-infected hamsters [29,32], the spleen of *L*. *infantum*-infected hamsters studied here showed low iNOS expression, which is produced by activated macrophage and that acts through nitric oxide (NO) synthesis to eliminate the parasite [33]. Besides, we observed a high arginase expression, an enzyme which is involved in the biosynthesis of crucial substrates, such as polyamines, that favors *Leishmania* growth [34]. With this, we believe that the failure to clear the infection in our model is related to the decrease in iNOS levels and increase in the arginase content. IFN-γ was highly expressed at 120 and 180 dpi in our infected hamsters studied, but apparently had no effect in activating macrophages, consequently no impact in parasite control. In the *L (L.) donovani*-infected hamster model, previously study showed that there is an impaired macrophage activation and an inability to control parasite replication despite a strong expression of Th1-like cytokines in the spleen [29]. Nevertheless, they showed an absence of iNOS expression, which is a critical antileishmanial effector. Therefore, the reduction of iNOS and the increase of arginase seem to be crucial to the uncontrolled parasite burden in this model.

At the end of the follow-up, the animals did not present yet disturbances in erythrocyte lineage. Nevertheless, lymphopenia, neutropenia, eosinopenia, and thrombocytopenia were observed. The hematological findings observed in infected hamsters at 120 and 180 dpi corroborate with blood cell disturbances found in humans and experimental models of VL [35,36]. Thrombocytopenia and leukopenia with marked neutropenia and eosinopenia are often found in human VL and are usually affected by the duration of infection [37]. In dogs, leukopenia due to lymphopenia and eosinopenia is associated with severe clinical manifestations [38]. In the golden hamster model, intraperitoneal *L*. *infantum*-infected animals had thrombocytopenia and leukopenia (eosinopenia and neutropenia) were also noted after 6 months of infection [30].

Regarding biochemical analyzes, serum changes in transaminase levels (ALT/AST) suggest possible liver dysfunction. Elevated ALT levels in VL are reported in humans, dogs, and hamsters [39,40, 30]. In VL, increased serum ALP levels in line with liver tissue damage or injury are observed [41].

Glucocorticoids (GCs), also known as stress hormones, regulates a wide variety of physiological events, mainly in the immune system, where they exert anti-inflammatory and immunosuppressive activities [42]. Plasma cortisol is the glucocorticoid hormone predominant in hamsters subjected to chronic stress [43]. In this work, male hamsters were chosen to avoid estrogen and other female hormones´ interference on cortisol production. The high systemic cortisol levels found provides insights to an endocrine imbalance due to *L*. *infantum* infection. Two studies evaluating hormonal changes in VL, conducted in Brazilian chronic VL patients, also reported a higher plasma cortisol levels. However, these authors did not determine the relationship between hormonal disturbances and clinical and laboratorial features of VL [18,19]. On the other hand, in *L*. *braziliensis* cutaneous leishmaniasis patients no changes in the cortisol levels were detected [44], pointing to the importance of the systemic infection to HPA disturbances. Besides, other systemic infections such as malaria and Chagas disease [14,15] also present a hyperactivation of HPA axis, supporting our results that VL is involved with altered endocrine status of the HPA axis by the raise of glucocorticoids circulating levels.

Currently, the most used methods to address immunological features in hamsters are RT-qPCR for the detection of immunity-related gene expression, which consists in a limitation for studies using this model [23,45]. Increased cytokine induction observed in the chronic phase of human VL [6] was also presented in our model.

In particular, IFN-γ levels increase dramatically over the course of infection, but the individual analysis of IFN-γ expression showed that this cytokine was strongly negative correlated with cortisol, similar to observed in *L*. *braziliensis* CL patients [44]. One explanation for this is that glucocorticoids induce a Th2 polarization by inhibiting IFN-γ signaling [46]. In fact, we noted a strong positive correlation between glucocorticoids and IL-10 and TGF-β, which can explain the fail to kill the protozoa. On the other hand, there is a robust positive association between cortisol and pro-esteroidogenic enzymes, IL-1β and IL-6, that can justify the increase in the cortisol levels.

IL-6, a pro-inflammatory cytokine highly expressed on active VL and related to severe disease [6,47], was elevated in this study at 120 and 180 dpi. Another pro-inflammatory cytokine, IL-1β, was strongly positively correlated to cortisol levels during the infection. Generally, GCs are produced by the adrenal glands through the action of ACTH on its receptor MC2R [42]. Nevertheless, alternative pathways of GC steroidogenesis may also occur in the context of some infectious diseases. This increase of pro-steroidogenic cytokines can participate in the augment of cortisol levels in the hamster model of VL. Nevertheless, it cannot rule out the role of other mechanisms associated with steroidogenesis, such as the presence of parasites in the adrenal glands. However, the presence of parasites in the adrenal gland was not a common finding in VL dogs since it was immunostained detected in only 8 out of 45 animals [48]. This influence of pro-inflammatory cytokines on the neuroendocrine system is widely discussed on experimental Chagas disease. A study reported high levels of GC in the serum of *T*. *cruzi*-infected mice in acute and chronic infection, concomitant to an increase in IL-6 and IL-1β systemic levels. These changes were also followed by a decrease in corticotropic release hormone (CRH), but no significant changes in adrenocorticotrophic hormone (ACTH) levels, indicating that IL-1β and/or IL-6 *per se* are directly involved in GC release by the adrenal gland [49]. This finding is reinforced by studies on *T*. *cruzi*-infected TNF receptor knock-out mice that showed high glucocorticoid levels in parallel to increased IL-6 and IL-1β levels [50].

Although VL patients presented high circulating levels of pro-inflammatory cytokines, as IL-6 and IFN-γ, it is common the concomitant high production of anti-inflammatory cytokines as a possible homeostatic mechanism to control persistent infection-induced inflammation [46]. The immune markers that help with *L*. *infantum* replication such as IL-10 and TGF-β [51,52] were elevated in the infected hamsters studied by us.

We also detected that cortisol was an important node in the network interaction in comparison to others VL parameters. The same phenomenon was observed in all times evaluated. The levels of cortisol showed to be positively correlated to parasite burden, biochemical parameters (AST/ALT/ALP) related to liver damage, as well pro-inflammatory cytokines *(*IL-6 and IL-1β), anti-inflammatory cytokines (IL-10 and TGF-β), and enzyme (arginase) that may favor the progression of infection. Nevertheless, markers related to disease control (IFN-γ and iNOS) were negatively correlated to cortisol, which suggests that cortisol could be involved in VL pathogenesis. Cortisol can be related to the down-regulation of iNOS expression during infection because this hormone can induce a transrepression of the transcription of this enzyme [53].

Data in the current report described the association between cortisol levels and clinical, parasitological, and immunological parameters that may lead to severity of experimental VL in a *L*. *infantum*-hamster model. These findings will serve as a basis for investigating a possible causal relationship between the cortisol production and parameters modulation that leads to VL progression. Further studies will be necessary to address if hypercorticoidism showed in *L*. *infantum*-hamster model of VL has a cause-and-effect relationship with the severity of the disease.

## Supporting information

S1 Table Hematological and biochemical follow-up evaluation of hamsters infected with *Leishmania (L*.*) infantum*. Abbreviation: mean corpuscular volume (MCV), mean corpuscular hemoglobin (MCH), mean corpuscular hemoglobin concentration (MCHC), alanine aminotransferase (ALT), aspartate aminotransferase (AST), alkaline phosphatase (ALP), total bilirubin (TBIL), albumin (ALB), days post-infection (dpi). a Values are represented by mean ± standard deviation; results of two independent experiments. b Value of significance p< 0.05.
(TIF)
