# Supplementary figures and images for: Increased levels of cortisol are associated with the severity of experimental visceral leishmaniasis in a Leishmania (L.) infantum-hamster model
Source: PLoS Negl Trop Dis. 2021 Nov 23;15(11):e0009987. doi: 10.1371/journal.pntd.0009987 (PMC8651114; doi:10.1371/journal.pntd.0009987)

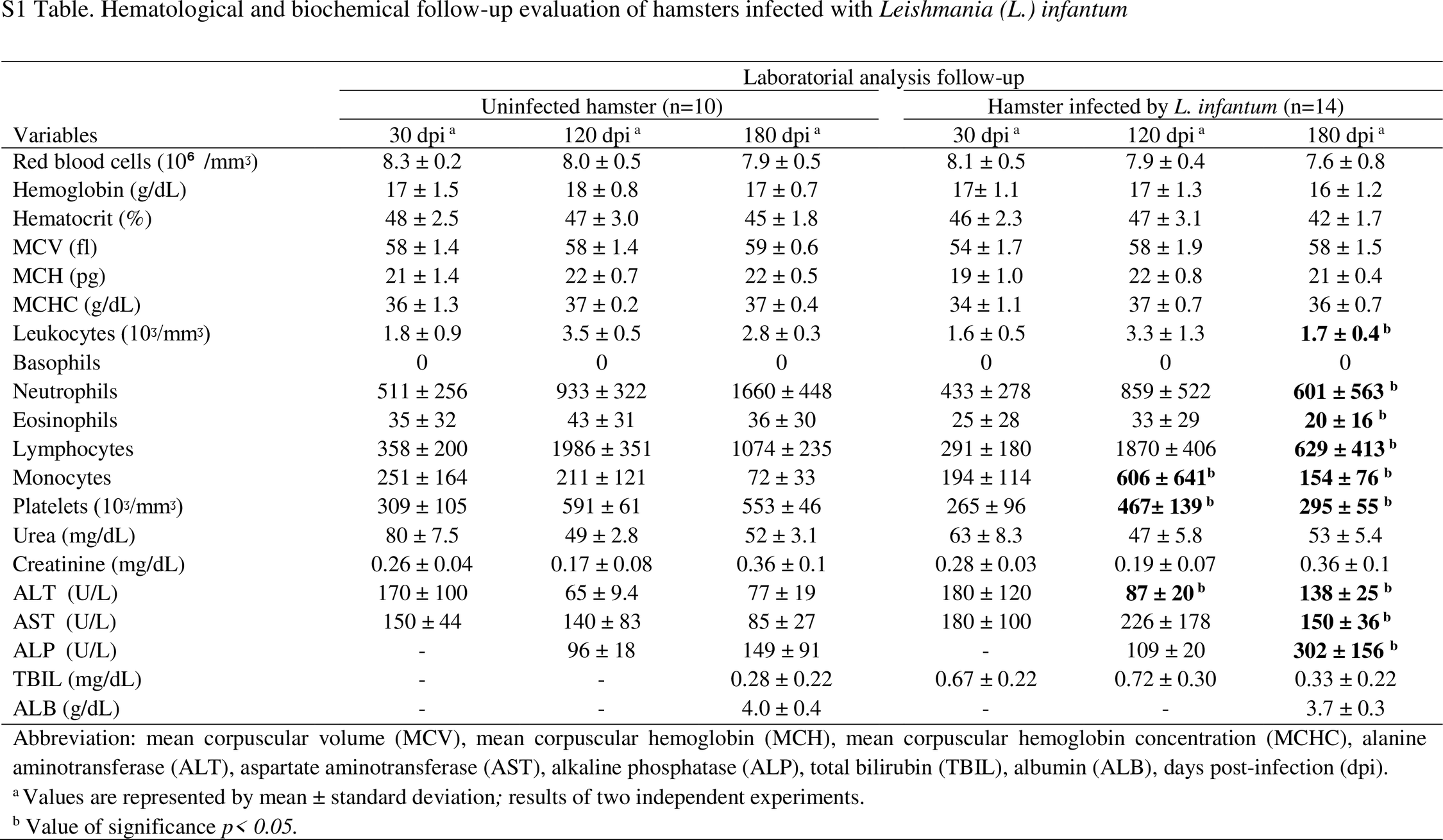

Supplement: S1 Table — Abbreviation: mean corpuscular volume (MCV), mean corpuscular hemoglobin (MCH), mean corpuscular hemoglobin concentration (MCHC), alanine aminotransferase (ALT), aspartate aminotransferase (AST), alkaline phosphatase (ALP), total bilirubin (TBIL), albumin (ALB), days post-infection (dpi). a Values are represented by mean ± standard deviation; results of two independent experiments. b Value of significance p< 0.05. (TIF) [file pntd.0009987.s001.tif]
